# Inclusion body myositis: Update on the diagnostic and therapeutic landscape

**DOI:** 10.3389/fneur.2022.1020113

**Published:** 2022-09-27

**Authors:** Elie Naddaf

**Affiliations:** Department of Neurology, Mayo Clinic, Rochester, MN, United States

**Keywords:** inclusion body myositis, idiopathic inflammatory myopathies, neurodegenerative diseases, aging, individualized medicine

## Abstract

Inclusion body myositis (IBM) is a progressive muscle disease affecting patients over the age of 40, with distinctive clinical and histopathological features. The typical clinical phenotype is characterized by prominent involvement of deep finger flexors and quadriceps muscles. Less common presentations include isolated dysphagia, asymptomatic hyper-CKemia, and axial or limb weakness beyond the typical pattern. IBM is associated with marked morbidity as majority of patients eventually become wheelchair dependent with limited use of their hands and marked dysphagia. Furthermore, IBM mildly affects longevity with aspiration pneumonia and respiratory complications being the most common cause of death. On muscle biopsy, IBM is characterized by a peculiar combination of endomysial inflammation, rimmed vacuoles, and protein aggregation. These histopathological features are reflective of the complexity of underlying disease mechanisms. No pharmacological treatment is yet available for IBM. Monitoring for swallowing and respiratory complications, exercise, and addressing mobility issues are the mainstay of management. Further research is needed to better understand disease pathogenesis and identify novel therapeutic targets.

## Introduction

Inclusion body myositis (IBM) is a sporadic muscle disease of aging, almost exclusively affecting individuals over the age of 40 (1). Traditionally, IBM is classified as an idiopathic inflammatory myopathy. However, the unique clinical phenotype, the peculiar combination of various histopathological findings, and the slowly progressive, treatment-refractory course, made IBM a hot debate topic regarding its pathogenesis and the best way to treat it. As the clinical and histopathological features are not universally present in all IBM patients, patients are often misdiagnosed, especially earlier in the disease course (2, 3). Depending on the prominent clinical and/or histopathological features in a particular patient, common misdiagnoses include polymyositis or other myositides, hereditary myopathy with rimmed vacuoles or other inherited myopathies, compressive mononeuropathies (especially ulnar neuropathy at the elbow or median neuropathy at the wrist) or radiculopathies (especially L3/4 radiculopathy), or a motor neuron disorder. Hereditary myopathies with rimmed vacuoles, associated with a systemic proteinopathy, are sometimes called hereditary IBM (hIBM). However, the term hIBM (vs. sporadic IBM or sIBM) may be misleading, as it implies it is the hereditary form of the same disease, while indeed these are two different diseases with different clinical phenotype, disease course, and patient demographics. As treatment, prognosis, and implications for other family members is widely variable between these various entities, misdiagnosis can have a major implication on patient care. Furthermore, the lack of a curative treatment often results in loss to follow up and consequently, lack of monitoring for disease complications and providing adequate supportive care. In this review article, we focus on addressing these diagnostic and therapeutic challenges in patients with IBM.

## Epidemiology and long-term outcomes

Most epidemiologic studies in IBM focused on estimating the incidence and prevalence of the disease. IBM affects males about twice as common as females, with a prevalence varying from 1 to 182 per million among those aged 50 and older (4–8). This variability in the reported prevalence is at least in part due to variability in case ascertainment methods and the used diagnostic criteria. Regarding associated conditions, patients with IBM are 2.7 times more likely to have a peripheral neuropathy, 6.2 times more likely to have Sjogren syndrome, and 3.9 times more likely to have hematologic malignancies, especially T-cell large granular lymphocytic leukemia, when compared to population controls (9). In contrast, there is no evidence for increased prevalence of neurodegenerative diseases or solid cancers in IBM population (9, 10). Given the predilection to indispensable muscles, IBM is associated with marked morbidity. The muscle weakness steadily progresses over time with a variable decline rate, although progression may be more pronounced earlier in the disease course (11, 12). Despite having predilection to certain muscles at earlier stages, any skeletal muscle can be affected at advanced stages. Almost all patients become wheelchair dependent within 20 years from onset, with a median time from symptom onset to wheelchair dependence about 10.5 years (7, 12, 13). Furthermore, dysphagia is highly prevalent in IBM, with aspiration pneumonia, in addition to respiratory complications of the disease, being the most common cause of death (9, 14–16). As a result, IBM is associated with a modest decrease in longevity, with a 10-year survival of 36–42% compared to 59% in population controls, and a mean age at death of 79.3 years compared to 83.6 in controls (8–10).

## Clinical presentation

**Figure 1:**
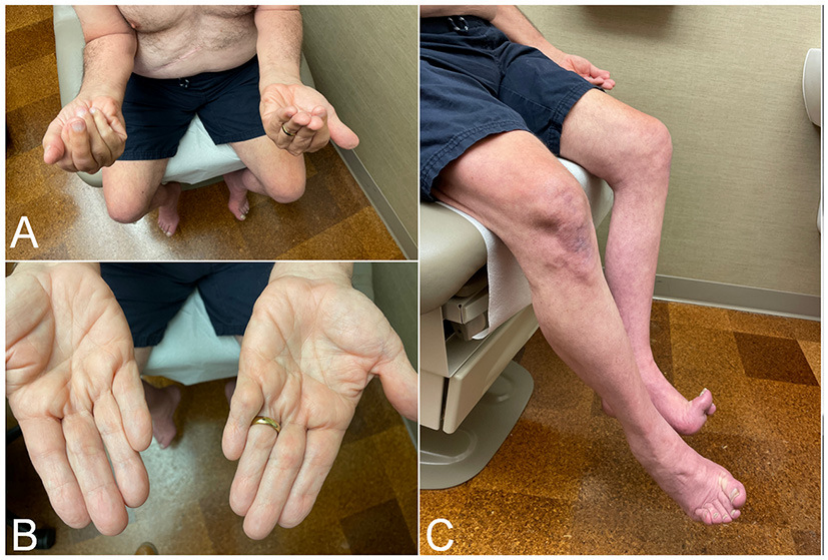
Clinical findings in patients with inclusion body myositis. **(A)** Patient attempting to make a fist, demonstrating bilateral finger flexion weakness most severely affecting flexion at the distal interphalangeal joint, worse on the left side. **(B)** Same patient as in **(A)**. Effacement of finger wrinkling over the palmar aspect of the interphalangeal joints, more pronounced on the left side, most noticeable over the distal interphalangeal joints. **(C)** Patient attempting knee extension, demonstrating bilateral quadriceps weakness, more severe on the left side where there is more noticeable thigh and leg muscle atrophy.

IBM is a slowly progressive disease, mimicking the clinical course of neurodegenerative diseases such as Parkinson's or Alzheimer's. Hence, presentation with rapidly progressive weakness, such as going from normal gait to needing a walking aid within a year from onset, should cast doubts about the diagnosis and prompt searching for alternative etiologies. IBM has predilection to finger flexors and knee extensors (Figure 1). As a result, patients most commonly present with either hand grip or lower limb weakness (e.g., difficulty with stairs, or difficult rising from a low seat), often asymmetric. This distinctive pattern of weakness, when present, strongly raises suspicion for the diagnosis. However, manual evaluation of the quadriceps strength can be challenging. Therefore, mild to moderate weakness of this muscle may be overlooked, and patients erroneously labeled as having intact strength despite reporting difficulty with their daily life activities. Manual testing of knee extension should be performed with the knee bent (e.g., 90 degrees), rather than fully extended or locked, so the knee mechanics are at the advantage of the examiner (17). Functional examination of the knee strength, such as kneeling on one knee then getting up without using the hands, should follow especially when weakness is not detected on manual testing. Examiner should keep in mind that patients may have difficulty performing this task without necessarily having quadriceps weakness, for instance in patients with hip or knee osteoarthritis or obesity.

About 14% of IBM patients have an atypical presentation beyond hand grip and quadriceps muscle weakness (3). Such patients may present with dysphagia, foot drop, proximal upper limb weakness, facial diplegia, axial weakness or head drop, or asymptomatic elevated creatine kinase level (hyper-CKemia) (3, 18–22). Respiratory insufficiency usually occurs at advanced stages of the disease. Patients with IBM do not typically have any significant cardiac muscle involvement, or extraskeletal manifestations of the disease.

## Diagnosis

Despite the distinctive clinical phenotype, a muscle biopsy remains the gold standard for diagnosis. It would be challenging to base the diagnosis solely on the quadriceps weakness, due to technical difficulty with manual muscle testing as mentioned above, and as the quadriceps may be involved to the same extent as hip flexors in some patients. While finger flexion weakness, more than shoulder abduction, can be more characteristic and easier to demonstrate, prominent finger flexion weakness can also be seen in other acquired (amyloidosis, sarcoidosis) and hereditary (especially myotonic dystrophy type 1) myopathies (23). Patients with atypical presentations pose additional diagnostic challenges with further delay in diagnosis (3). Therefore, clinical-pathological correlation remains crucial, and all widely-used diagnostic criteria require fulfillment of certain muscle biopsy features (1, 24).

### Electrodiagnostic testing

Nerve conduction studies and electromyography (EMG) help determining the nature of the process underlying the patient's reported weakness: myopathic vs. neuropathic, and rule out a motor neuron disorder or multiple mononeuropathies that can have similar presentation. Furthermore, EMG findings are taken in consideration when selecting a target for a muscle biopsy. Nerve conduction studies are usually within normal limits, or may detect a superimposed length-dependent peripheral neuropathy. Needle EMG typically demonstrates early recruitment of short duration, low amplitude, complex, motor unit potentials (MUP), with fibrillation potentials in almost all patients (25). Up to a third of patients may demonstrate myotonic or myotonic-like discharges (3, 25, 26). The findings are usually more prominent in weaker muscles, such as flexor digitorum profundus and quadriceps. Furthermore, mixed short and long duration MUP, within the same muscle, are often encountered in IBM (3, 25). In such patients, the short MUPs may be overlooked, and the patient may get erroneously diagnosed with a neuropathic process such as an anterior interosseous neuropathy or L3/4 radiculopathy. The long duration MUPs in IBM are often complex in morphology, mimicking a subacute neuropathic process (25). Similar to EMG findings, neuropathic changes (denervation atrophy and/or reinnervation) are seen in vast majority of muscle biopsies from patients with IBM (27).

### Muscle biopsy

**Figure 2:**
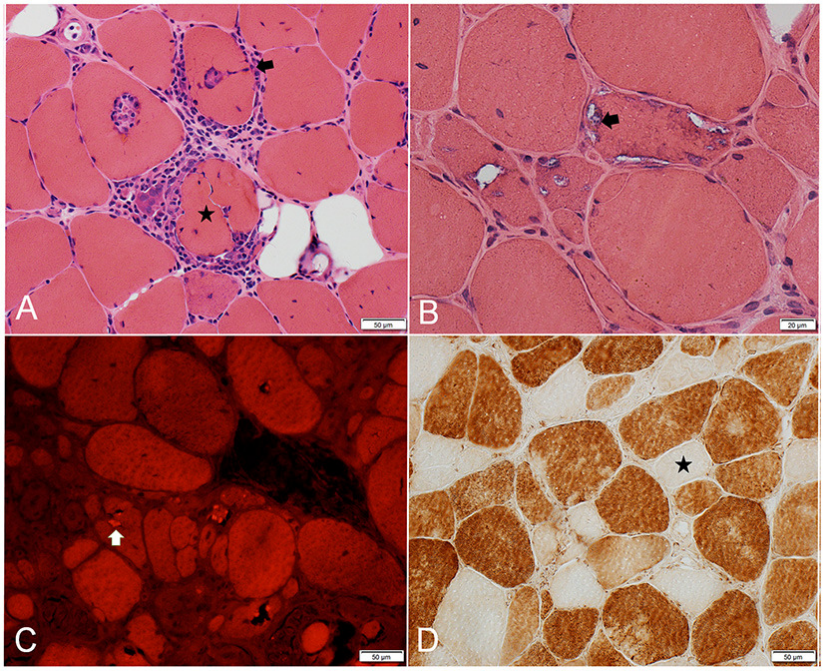
Frozen muscle sections from patients with inclusion body myositis. **(A)** H&E stain: inflammatory cells surrounding, and focally invading (arrow) muscle fibers. One muscle fiber (star) is completely split apart by inflammatory cells. **(B)** H&E stain: 3 adjacent muscle fibers harboring multiple vacuoles rimmed by a membranous material (example: arrow). **(C)** Congo Red stain viewed under Rhodamine optics: several fibers display one or more congophilic (bright red) inclusions (example: arrow). **(D)** Cytochrome C oxidase reaction: several fibers are devoid of enzyme reactivity (example: star).

Our understanding of IBM pathogenesis stemmed from the description of its peculiar histopathological findings (Figure 2). The three canonical features of IBM include: endomysial inflammation, where inflammatory cells surround and invade non-necrotic muscle fibers, also known as autoaggressive inflammation; the presence of rimmed vacuoles; and protein aggregation as witnessed by the accumulation of congophilic deposits and 15/18 nm filaments (tubulofilaments) on electron microscopy (EM) (28, 29). In order to establish the diagnosis of IBM on histological grounds (clinico-pathologically defined IBM), the three canonical features have to be present, in addition to fulfilling clinical and laboratory criteria (1). However, up to 25% of patients with clinical features of IBM do not have rimmed vacuoles or congophilic deposits on biopsy, which results in the erroneous diagnosis of “polymyositis” or “myositis not otherwise specified,” and unnecessary treatment with corticosteroids or other immunosuppressants (2, 30). Furthermore, the lack of congophilic deposits or tubulofilaments was the most common reason why patients with IBM failed to fulfill various diagnostic criteria in one study (31). However, Congo red stain and EM were only performed on a small proportion of patients. Moreover, EM is not widely available for clinical use. In our Muscle laboratory at Mayo clinic, Congo red staining is routinely performed on all muscle biopsies. Slides are reviewed under rhodamine optics, rather than polarized light, which is more sensitive for the detection of amyloid deposits (32). Detecting protein aggregates by alternative methods, such as TDP43 and p62 by immunohistochemistry, can help further increasing the diagnostic yield of a muscle biopsy (33, 34).

The most common muscle biopsy findings in IBM are the increased sarcolemmal expression of MHC class I, detected by immunohistochemistry, and the presence of cytochrome c oxidase negative fibers, both of which are present in almost all IBM muscle biopsies (Figure 2D) (30, 35, 36). Upregulation of MHC-I expression lacks specificity as it can be seen in other immune-mediated and sometimes inherited myopathies, limiting its diagnostic value. Combining MHC-I with MHC-II is reported to increase specificity to for the diagnosis of an inflammatory myopathy, however, MHC-II staining on muscle is not widely available yet (37). The presence of significant mitochondrial abnormalities in muscle specimens demonstrating endomysial inflammation should strongly raise suspicion for IBM, with one study reporting a 100% sensitivity and 74% specificity in this context (35). Patients with endomysial inflammation and prominent mitochondrial dysfunction, but without rimmed vacuoles or protein aggregates, are sometimes referred to as having “polymyositis with mitochondrial pathology” (PM-Mito) (38). It remains debatable whether this should be considered a separate entity, given the high prevalence of mitochondrial abnormalities in IBM and as a significant proportion of these patients are eventually diagnosed with IBM (39–41). A less common muscle biopsy finding in IBM is the presence of granulomas. IBM and sarcoid myopathy are the most common diagnoses in patients with granulomatous myositis on muscle biopsy (42, 43). Lastly, a component of denervation atrophy is seen in majority of biopsies, manifesting with groups of atrophic angulated fibers overreacting to non-specific esterase (25, 27).

Selecting the target for a muscle biopsy is of utmost importance. It is preferable to choose a clinically-affected muscle, where the weakness is of moderate severity (21). This would limit the chances of a false negative (normal or minimally affected muscle) or non-diagnostic endstage muscle (severe weakness). Using EMG (muscle with spontaneous activity) and muscle imaging data when available, can help optimize the yield of a muscle biopsy. Many institutions only perform muscle biopsies from the quadriceps, especially when performing a punch biopsy. Luckily, the quadriceps is commonly involved in IBM but not all patients have quadriceps involvement at presentation. One caveat with the quadriceps muscle is that the four heads are not usually equally affected. Hence, correlation with clinical examination, electrodiagnostic testing, and imaging would ensure proper selection of biopsy site. Lastly, muscle involvement may be asymmetric in IBM, which should be taken in consideration, especially when using EMG for muscle selection, as it is typically performed on one side saving the contralateral side for biopsy. Occasionally, patients may require a repeat muscle biopsy to establish the diagnosis (3, 21).

### Cytosolic 5'-nucelotidase antibodies

Antibodies against cytosolic 5'-nucleotidase 1A (cN-1A) are the only available serum diagnostic test for inclusion body myositis (44, 45). Overall, the sensitivity is limited, around 30–50%, when using ELISA, which is the most commonly used platform for commercial testing (46). Specificity is high, usually more than 90% (44, 45). However, it is much lower in patients with other connective tissue diseases, such as systemic lupus erythematous, Sjögren syndrome, or dermatomyositis, as up to a third of these patients may have positive cN-1A antibodies and not have IBM (47, 48). Given the challenges mentioned above and the variability in the used detection methods, cN-1A results should be cautiously interpreted in light of the patient's clinical and histopathological findings.

### Muscle MRI

**Figure 3:**
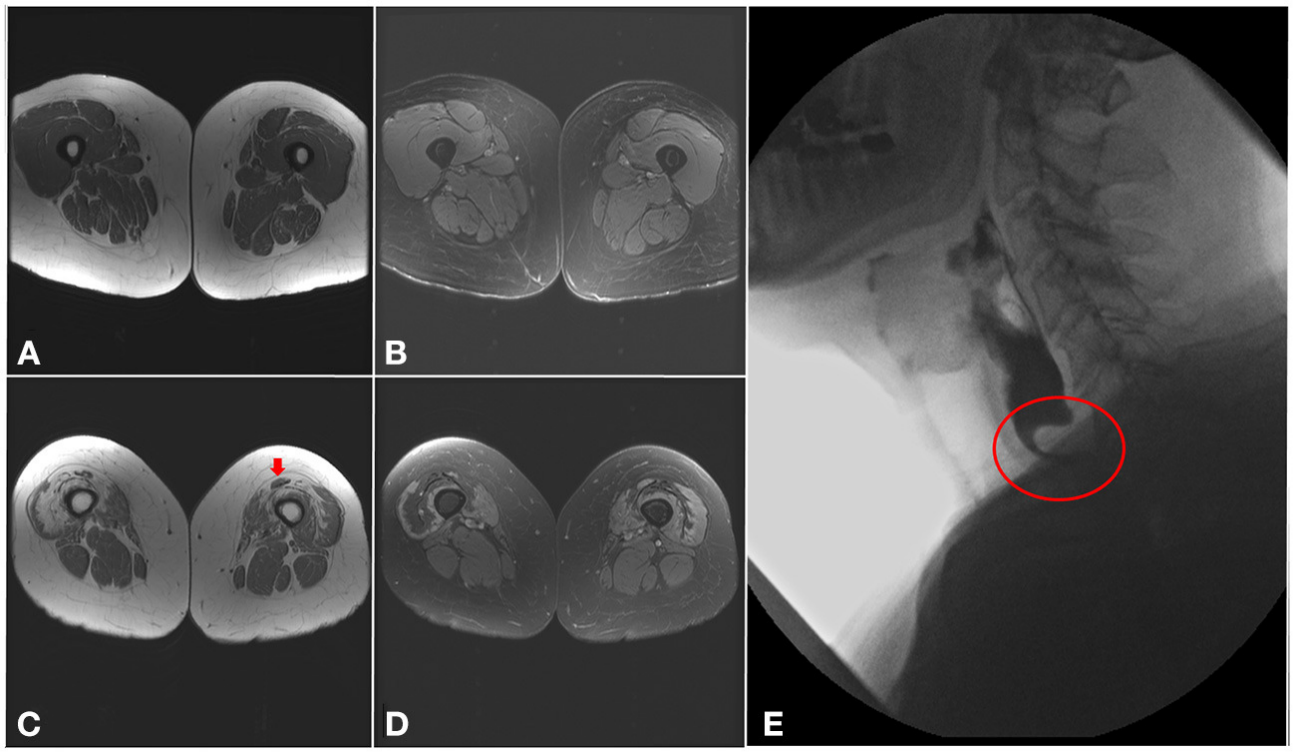
Imaging findings in inclusion body myositis. **(A–D)** MRI of the thighs from a patient with IBM. Axial T1 images **(A,C)** showing a proximal-to-distal gradient, with preservation of proximal segments **(A)** and fatty infiltration of the distal vastus medialis and lateralis bilaterally **(C)**, more pronounced in the right lower limb, with relative sparing of the rectus femoris (arrow). Axial T2 images **(B,D)** demonstrating sparing of the proximal segments of the quadriceps muscles **(B)**, and only mild T2 hyperintensity surrounding areas of fatty infiltration distally **(D)**. **(E)** Barium swallow demonstrating a cricopharyngeal bar with severe (more than 75%) luminal narrowing (circle).

Muscle MRI findings in IBM commonly follow the same clinical pattern with preferential involvement, sometimes asymmetrically, of finger flexors, mainly flexor digitorum profundus, and quadriceps. In the quadriceps, the rectus femoris is usually spared and there is a proximal-to-distal gradient, with more pronounced fatty infiltration near the knee (Figure 3) (49, 50). In the legs, the medial gastrocnemius is the most involved with sparing of the tibialis posterior and soleus muscles (51, 52). In contrast to other inflammatory myopathies, fatty infiltration is typically more prominent than increased T2 signal (edema) in patients with IBM. Nevertheless, the use of muscle MRI as a diagnostic tool in inflammatory myopathies remains limited (53). However, this classic MRI pattern should raise suspicion for IBM even if the diagnosis was not considered on clinical grounds, such as in patients with isolated hyper-CKemia or with atypical disease presentation (3).

### Other blood tests

Creatine kinase (CK) level is elevated in 75−80% of patients, typically < 15 times upper limit of normal (22, 25). Higher CK levels should cast doubt about the diagnosis and prompt searching for alternative acquired or inherited etiologies.

When screened, some IBM patients may have clonal expansion of a large granular T cells (T-LGL) population, and rarely T-LGL leukemia (9, 54). Differentiating T-LGL clonal expansion from T-LGL leukemia and the best therapeutic approach for the latter remain a topic of debate (55, 56). Therefore, from hematological perspective, routine screening for T-LGL leukemia is not recommended. One practical approach would be to obtain a CBC and a peripheral smear. The presence of lymphocytosis, cytopenias, and/or large granular T cells should prompt further investigation *via* T-cell receptor gene rearrangement and/or flow cytometry, and referral to Hematology. Nevertheless, from neurological perspective, the utility of detecting a T-LGL clone as a diagnostic tool for IBM has not been fully investigated.

### Correlation of laboratory and clinical findings

Regarding muscle biopsy findings, overall, there is no clear or significant correlation between clinical findings and muscle histopathological features (27). In a recent study, we evaluated the correlation of various muscle biopsy findings with clinical variables including quadriceps strength, summated strength score, modified Rankin scale, severity of dysphagia, and baseline characteristics such as age at biopsy and disease duration (27). The main clinical variable that had a strong [Kendal tau correlation coefficient (k) of 0.62] and statistically-significant correlation with endomysial inflammation was the severity of dysphagia, evaluated by a formal swallow evaluation. This is intriguing as dysphagia is the most anecdotally-reported manifestation of IBM with some response to immunotherapy (57, 58). The most significant correlation with quadriceps strength was the increased endomysial connective tissue, reflective of the chronic loss of muscle fibers (27). Interestingly, modified Rankin scale was inversely (k = 0.39) correlated with inflammation (27). Rimmed vacuoles and congophilic deposits had no notable (k < 2) or statistically significant correlation with any clinical variable (27).

Regarding cN-1A antibodies, there are conflicting results about the association of cN-1A seropositivity with more prominent (degree of severity) or more frequent (present or not) dysphagia, which can be in part due to the variability in the methods used (27, 59–64). Similarly, there are varying results regarding the association of seropositivity with the severity of limb muscle weakness or pattern of weakness (27, 59, 62, 63). Taken altogether, cN-1A seropositive patients may be more likely to have slightly more pronounced dysphagia and/or muscle weakness. However, if such association is indeed present, it is modest at best, and not sufficient to use the antibody to predict severity or disease phenotype in clinical practice.

Lastly, Ck levels do not correlate with clinical findings in IBM (22, 27). Hence, decreased CK levels should not be used as evidence for response to immunotherapy and a reason to keep patients on such treatments.

## Disease mechanisms

Traditionally, IBM has been considered an inflammatory muscle disease. The main support for the inflammatory hypothesis is the robust inflammatory infiltrate on muscle biopsy, where inflammatory cells, namely cytotoxic CD8+T cells, surround and invade non-necrotic muscle fibers (28). These T cells are clonally restricted, antigen driven, and express markers of high differentiation such as CD57 and KLRG1 (65–70). Furthermore, there is a strong association between IBM and HLA-DRB1 genes (71–73). However, the refractoriness to immunosuppressive therapy, the lingering disease course, the age of the at-risk population, and the male predominance would all be atypical for an inflammatory disease. In addition to the lingering course and the characteristics of the population at risk, IBM shares common pathological features with other neurodegenerative diseases, namely the accumulation of rimmed vacuoles and protein aggregates such as amyloid-β precursor protein and amyloid- β, as seen in Alzheimer's disease, and p62 and Tar-DNA binding protein 43 (TDP-43) as seen in amyotrophic lateral sclerosis and frontotemporal dementia (29, 74–76). While the role of amyloid deposits in IBM pathogenesis remains uncertain, the accumulation of TDP-43+ inclusions in the sarcoplasm of IBM patients is accompanied by TDP-43 nuclear depletion, resulting in loss of TDP-43 splicing repression of non-conserved cryptic exons, a feature seen in amyotrophic lateral sclerosis and frontotemporal dementia (77). Several non-inflammatory pathways have been briefly described in IBM, although the exact level of dysfunction in each pathway remains poorly defined. Those include disrupted protein homeostasis, excessive and/or impaired autophagy, mitochondrial dysfunction, oxidative stress, disrupted calcium homeostasis, ER stress and extracellular matrix involvement (78–81). A more detailed review of these pathways is out of the scope of this review article, some of them are nicely summarized in (82, 83).

## Treatment

Despite the lack of pharmacological treatment for IBM, there are several items that need to be addressed on a regular basis: swallowing, respiratory function, muscle weakness and mobility.

Dysphagia is often overlooked despite aspiration pneumonia being the most common cause of death. Screening should probably be performed on an annual basis, at least by obtaining a detailed history and keeping a low threshold for a referral to a speech pathologist for a formal evaluation. Questionnaires, such as the “Eating Assessment Tool” (EAT) can be used (84). When present, the dysphagia may be due to a combination of obstruction and weakness. The cricopharyngeus muscle can scar, which results in luminal obstruction, known as a cricopharyngeal band or bar (Figure 3E) (3, 85). If the obstruction is significant, endoscopic dilation or a cricopharyngeal myotomy could be considered. For the oropharyngeal weakness, treatment consists mostly of adaptive strategies such as eating specific consistencies or volumes to avoid aspiration. There is anecdotal evidence for improved dysphagia with immunotherapy, namely IVIG, which may provide temporary relief in selected cases, however there is no evidence that IVIG changes the overall disease course (57, 86, 87).

Similar to dysphagia, respiratory failure is a major source of morbidity and mortality in IBM. It is important to screen *via* a detailed questionnaire and to have a low threshold to perform additional testing, such as overnight oximetry, pulmonary function tests, or a sleep study, and to refer the patient to a sleep medicine specialist when appropriate. While dysphagia can occur at any disease stage, respiratory failure usually occurs at advanced stages.

Routine evaluation by physical medicine and rehabilitation team is often needed. This includes offering adaptive strategies or assistive devices (walker, bracing, wheelchair etc.) to help with hand dexterity and mobility, evaluating for safety and fall prevention, and providing an exercise program. Exercise, especially resistance training, may help preserving or even improving muscle strength in patients with IBM (88, 89).

There is no evidence-based pharmacological therapy for IBM. Yet, prednisone remains one of the most commonly prescribed drugs in IBM. To note, treatment with corticosteroids could potentially be harmful, although this has not been established with certainty either. In one study, Benveniste et al. reported that treated IBM patients (with corticosteroids or other immunosuppressants) needed a walking aid sooner than untreated patients (12). Comparison of baseline characteristics between treated and untreated groups was not available. In another study, there was a separation in survival curves for IBM patients treated with corticosteroids (lower survival) compared with untreated IBM patients, with no difference in age, sex, the presence of dysphagia, gait difficulty or follow-up duration between the two groups (9). Although confounding factors could not be excluded in both study, it'd be best to avoid empiric treatment with corticosteroids or other immunosuppressants given the lack of evidence to support their efficacy in IBM.

In addition to prednisone and IVIG, other agents targeting the immune system that have been tried in IBM include: azathioprine, methotrexate, antithymotcyte globulin, etanercept, anakinra, alemtuzumab, natalizumab and IFNβ1A (90). Furthermore, agents targeting non-inflammatory pathways included: lithium, oxandrolone, follistatin gene therapy, bimagrumab, arimoclomol, and rapamycin. Lithium was considered because it inhibits glycogen synthase kinase-3 (GSK). GSK is involved in various cell processes, such as autophagy, cell survival/differentiation, and cell cycle regulation, and has been associated with hyperphosphorylation of tau proteins [reviewed in (91)]. Oxandrolone (synthetic androgen), follistatin (myostatin inhibitor) gene therapy, and bimagrumab (myostatin inhibitor) have been considered as enhancers of skeletal muscle mass development (92–94). Arimoclomol is a heat shock protein inducer and was considered for improving protein homeostasis in stressed cells (95, 96). Rapamycin (sirolimus) was considered due to its immunosuppressive effect and ability to enhance autophagy by inhibiting the mammalian target of rapamycin (mTOR) that is an autophagy inhibitor (97). Phase 2 trial was recently completed and the primary outcome was reportedly not met. However, the full results for this study and many of the mentioned trials have not been published yet in peer-reviewed journals.

There are two main ongoing trials in IBM: Sirolimus and ABC008. Sirolimus is currently being tested in a multicenter phase 3 trial (https://clinicaltrials.gov/ct2/show/NCT04789070?recrs=abdf&cond=inclusion$+$body$+$myositis&draw=6&rank=5). ABC008 is a monoclonal antibody targeting KLRG1 receptor, which selectively depletes highly differentiated cytotoxic T cells (https://clinicaltrials.gov/ct2/show/NCT04659031). In an IBM xenograft model, human T cells were depleted from xenografts in 4 mice by treatment with a CD3 monoclonal antibody (OKT3), and compared to 4 untreated xenografts (77). MHC1 expression was subsequently reduced in the treated samples, however, rimmed vacuoles and loss of TDP-43 function persisted when evaluated at 2- and 4-months post treatment (77). The small sample size, the low percentage of muscle fibers displaying rimmed vacuoles, and the limited follow up time were acknowledged as study limitations. It remained unclear if the persistently detected myodegenerative changes reflected ongoing disease activity vs. residua from previous damage. Whether KLRG1+-T- cell depletion will halt disease progression is yet to be determined.

## Current challenges and future directions

The most critical unmet need in IBM remains the lack of an effective treatment. This is due to several factors, especially poor understanding of the underlying pathogenesis. The complexity of IBM histopathology and disease mechanisms sparked an ongoing debate on whether the disease is primarily inflammatory or neurodegenerative in nature (98). However, determining which is the cause vs. the consequence may not be the most crucial factor to find effective treatments. Similar to other chronic disorders, the various involved immune and non-immune pathways likely form intertwined, irreversible vicious circles, that are sustained over time (99). Further research is needed to better understand the relationship between the innate immune system and neurodegeneration first, define the exact level of dysfunction in the invoked pathways in IBM, and then identify novel therapeutic targets that would help break that destructive loop. Whether systems biology approaches will help define involved pathways on an individual patient level is yet to be determined. Biological and technical variabilities remain a challenge.

From clinical trial design perspective, the main challenges are inherent to IBM's clinical heterogeneity, relative rarity, and slowly progressive course. The clinical phenotype is variable at early stages of the disease, the period where it would be ideal to intervene, whereas patients typically converge into the classic phenotype at more advanced stages (3). Hence, earlier in the disease course, the disability profile can be mostly driven by the difficulty walking, the limited use of the upper limb, the difficulty swallowing or any combination thereof. Furthermore, the weakness is commonly asymmetric differentially affecting the right and left side. This clinical heterogeneity made it challenging to have an outcome measure with sound reliability and content validity. For instance, commonly used lower limb-focused outcome measures (6-min walk distance, quadriceps strength and thigh muscle volume) may have limited validity in patients whose disability is mostly driven by upper limb or swallowing dysfunction (94). In addition to the limited sampling frame in general due to disease rarity, powering clinical trials to detect differences among patient subgroups, such as early vs. late in the disease course, disability profile, race, or sex, would affect feasibility, especially that all currently considered or in-trial drugs are expected to have, at best, a stabilizing or modest effect. Furthermore, the slowly progressive disease course makes it challenging to detect a treatment effect within a 6-to-12-month trial period. It is important to note that the traditional classification of IBM as an idiopathic inflammatory disease has indirectly affected expectations from clinical trials, as “myositis” would be expected to markedly improve or resolve for a drug to be deemed effective.

In the era of personalized medicine, individualized outcome measures and clinical trial designs offer an innovative approach to address such difficulties. The n-of-1 trial design concept is intriguing and not yet explored in IBM. N-of-1 trials allow to evaluate treatment response on an individual level using a double-blind, randomized, multiple crossover design, following the same quality standards as traditional trials (100). Results from multiple individuals could be aggregated and analyzed at a group level. The n-of-1 design is best suited for the investigation of chronic or progressive conditions, and one third of such trials have been conducted for neurological disorders (101). Despite challenges, especially logistical and related to data analysis, the n-of-1 trial design is akin to the concept of individualized medicine, and may allow to determine the best treatment regimen for a particular patient, and limit the time spent on suboptimal and/or expensive drugs approved based on results from larger traditional clinical trials (100).

Lastly, the main challenge of translating basic science findings into drug development and clinical trials is the limited availability for disease models. Hereditary inclusion body myopathy (hIBM) with frontotemporal degeneration (IBMPFD) models due to mutations in *VCP* have been used (102–104). However, these models had major limitations, especially that systemic proteinopathies, such as VCP-myopathy, are clinically distinct from IBM as discussed in the introduction. The IBM xenografts remain thus far the closest to recapitulate disease pathology but preclude functional and behavioral evaluation (77).

IBM is a chronic progressive disease of aging with variable disease onset, decline rate, and disability profile, at least in earlier stages of the disease. A more precise definition of involved, especially non-inflammatory, pathways, more reliable and valid outcome measures, and more variety in identified therapeutic targets are essential in order to make significant advancement in treating the disease.

## Author contributions

EN: design and conceptualization of the work, and writing of the manuscript.

## Conflict of interest

The author declares that the research was conducted in the absence of any commercial or financial relationships that could be construed as a potential conflict of interest.

## Publisher's note

All claims expressed in this article are solely those of the authors and do not necessarily represent those of their affiliated organizations, or those of the publisher, the editors and the reviewers. Any product that may be evaluated in this article, or claim that may be made by its manufacturer, is not guaranteed or endorsed by the publisher.
